# Separation of Minor Actinides from High-Level Liquid Waste Using Novel Silica-Based Butyl-BTP Adsorbents

**DOI:** 10.3390/toxics10120741

**Published:** 2022-11-30

**Authors:** Tianjiao Jiang, Shunyan Ning, Tao Yu, Ji Wang, Yuezhou Wei, Yan Wu, Hui He, Fangqiang Chen, Qingsong Wang

**Affiliations:** 1 School of Nuclear Science and Technology, University of South China, Hengyang 421001, China; 2 School of Nuclear Science and Engineering, Shanghai Jiao Tong University, Shanghai 200240, China; 3 Department of Radiochemistry, China Institute of Atomic Energy, Beijing 102413, China; 4 Nuclear and Radiation Safety Center, MEE, Beijing 102400, China

**Keywords:** high-level liquid waste, minor actinides, separation, R-BTP adsorbent, hot test

## Abstract

To separate the long-lived minor actinides (MA = Am, Cm) from high-level liquid waste (HLLW), we have been studying an advanced separation process via selective adsorption that uses minimal amounts of organic solvent and compact equipment. The process consists of two separation columns packed with a CMPO (octyl(phenyl)-N,N-diisobutylcarbamoyl-methyl phosphine oxide) adsorbent for elemental group separation and a soft-donor named the R-BTP (2,6-bis-(5,6-dialkyl-1,2,4-triazine-3-yl) pyridine) adsorbent for the isolation of MA from lanthanides (Ln). In this work, the effects of nitrate ion (NO_3_^−^) on the adsorption behavior of Am(III) and a typical fission product Ln(III) onto the *iso*Bu-BTP/SiO_2_-P adsorbent were studied experimentally. Then, the desorption properties of the adsorbed element were examined using different eluting agents. A hot test for the separation of MA from the fission product Ln in a genuine MA containing effluent from the irradiated MOX-fuel treatment process was carried out using a *n*Bu-BTP/SiO_2_-P packed column. It was found that the separation factor between Am(III) and Ln(III)-FP is over 100 in the measured 0.5–4 M NO_3_^−^. The adsorbed elements could be effectively eluted off using a complexing agent such as DTPA or pure water. Complete separation between MA and Ln was achieved in the column results, indicating that the proposed MA separation process is feasible in principle.

## 1. Introduction

**Figure 1:**
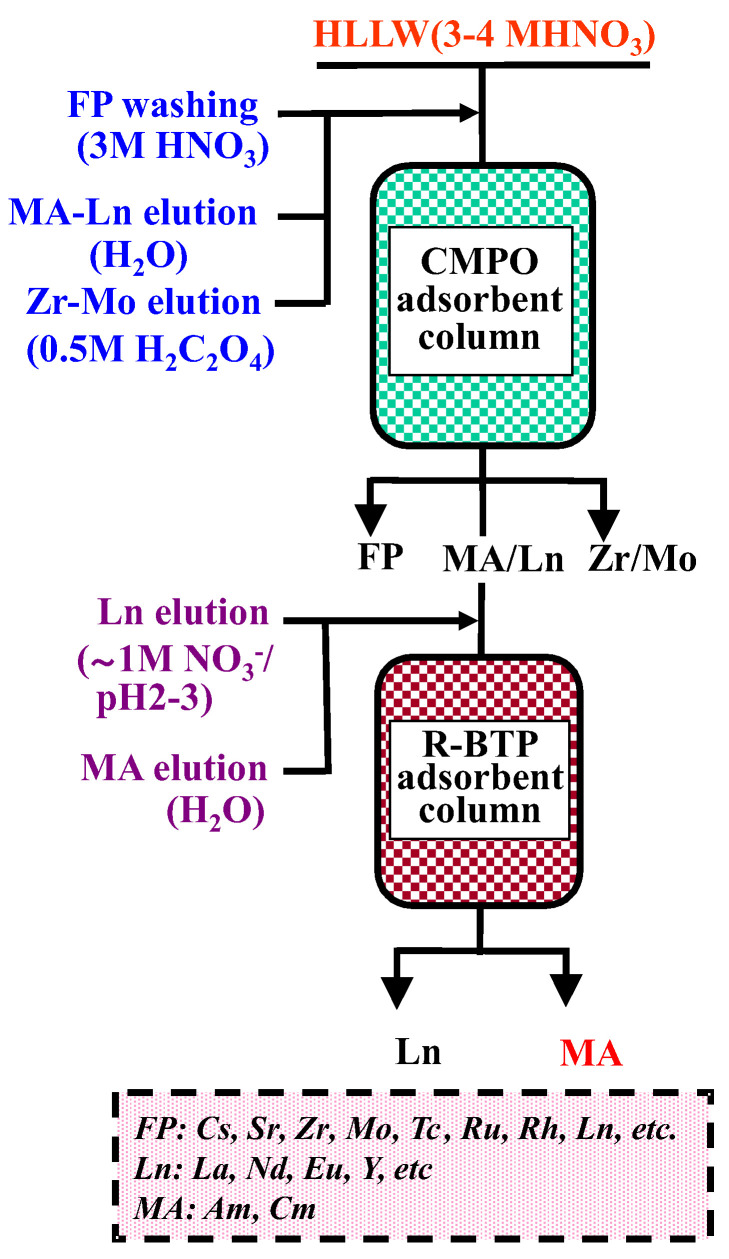
Schematic flowsheet of the MA separation process from HLLW using the adsorption method [11].

As a final disposal method for the high-level liquid waste (HLLW) generated by spent fuel reprocessing, as with the PUREX process, the geological disposal of vitrified HLLW has been proposed and investigated worldwide. Minor actinides (MAs; Am, Cm, etc.), which are alpha emitters with long half-lives (e.g., ^241^Am_t1/2_ = 432.2y, ^243^Am_t1/2_ = 7.38 × 103 y), contribute most of the long-term radiotoxicity of high-level liquid waste [1,2]. The efficient separation of MAs is of great significance for the final geological disposal of the HLLW and is also an essential component of the partition and transmutation (P&T) strategy to transmute the long-lived nuclides into short-lived or stable nuclides [3,4,5]. The fission products lanthanides (Lns) are much more abundant than MAs in HLLW and are neutron poisons, so they need to be separated before MA transmutation. Since Ln(III) possesses very similar chemical properties to MA(III) in solution, the selective separation of MA(III) from Ln(III) has become one of the most challenging technical problems in HLLW partitioning [3,4,5,6,7]. From the viewpoints of minimizing the long-term radiotoxic risk to the environment and facilitating the P&T strategy, the separation of the long-lived minor actinides (Am, Cm) from HLLW is an important task. For this purpose, we have been studying an advanced separation process involving a selective adsorption technology that uses a minimal organic solvent and compact equipment [2,3,5,8,9,10]. The schematic flowsheet of this process is shown in Figure 1 [11]. The process is constituted by two separation columns packed with CMPO (octyl(phenyl)-N,N-diisobutylcarbamoyl-methyl phosphine oxide) and R-BTP (2,6-bis-(5,6-dialkyl-1,2,4-triazine-3-yl)pyridine, R = alkyl group) adsorbents, respectively. In the first column, the elements contained in the HLLW can be partitioned into (a) non-adsorptive fission products (FP), (b) MA-Ln, and (c) Zr-Mo, according to their different adsorption and elution properties with CMPO and the eluents. The MA-Ln effluent is then supplied to the second column packed with the R-BTP adsorbent, where the MA is expected to be selectively isolated from the Ln fission products, since the trivalent actinides have much stronger adsorbability onto R-BTP than the trivalent lanthanides [4,5,6,7]. For this process, we synthesized some new silica-based adsorbents by impregnating CMPO or R-BTP into a styrene–divinylbenzene copolymer, which was immobilized in porous silica particles with a mean diameter of 60 μm (SiO_2_-P). Compared to conventional polymeric matrix adsorbents, these highly porous silica-based adsorbents with a small particle size have faster diffusion kinetics with a relatively lower pressure drop in packed columns, making them suitable for efficient chromatographic separation [8,9]. In our previous work, it was demonstrated that Am together with the fission product Ln can be successfully separated from other FPs contained in a genuine HLLW solution, which was generated from an irradiated MOX fuel treatment process using a CMPO/SiO_2_-P packed column [12].

It is well known that the mutual separation of the trivalent actinides with 5f-orbital electrons and the lanthanides with 4f-orbital electrons is one of the most difficult tasks in the nuclear fuel cycle due to their very closed ionic size and similar chemical properties. At the end of last century, Kolarik et al. reported that a multidentate nitrogen donor ligand, 2,6-bistriazinylpyridine (BTP), exhibits much higher extraction selectivity for Am(III) than Ln(III) [6,7]. Since 2000, we have synthesized some new R-BTP ligands with different alkyl groups (C = 1–8) and studied their separation behavior for MA and Ln and their chemical and radiation stability in nitric acid solutions [13,14,15]. As a result, the adsorption affinity and stability strongly depend on the structure of the alkyl group in 2,6-bis-(5,6-dibutyl-1,2,4-triazine-3-yl)pyridine adsorbent. It was found that Bu-BTP/SiO_2_-P showed significantly higher adsorption ability and selectivity for Am(III) than Ln(III). The separation experiment results for a simulated HLLW containing trace amounts of ^243^Am(III) and typical Ln(III) using a *n*Bu-BTP/SiO_2_-P packed column demonstrated that the Am(III) was completely separated from the Ln(III) [15].

Since Am(III) is present as a cationic ion in weak nitric acid solutions and R-BTP is a neutral compound, their coordination reaction requires anionic nitrate to form a stably neutral complex. In this work, we investigate the effects of the nitrate ion (NO_3_^−^) concentration on the adsorption behavior of Am(III) and the typical fission product Ln(III) on the Bu-BTP/SiO_2_-P adsorbent. Then, the desorption(elution) properties of the adsorbed element are examined using different eluting agents. A small-scale column separation experiment using a simulated high-level waste solution and a hot test for a genuine MA-containing effluent obtained from an irradiated MOX-fuel treatment process are carried out to verify the feasibility of the proposed separation process.

## 2. Materials and Experiment Methods

### 2.1. Reagents

^241^Am(III) and ^152^Eu(III) were obtained from a stock solution from the China Institute of Atomic Energy (CIAE). Commercially available Ln(NO_3_)_3_•nH_2_O at 99.9% purity was used to prepare the adsorption solution. The other agents used were of analytical grade. The aqueous solutions were prepared with deionized water.

### 2.2. Synthesis and Characterization of Materials

**Figure 2:**
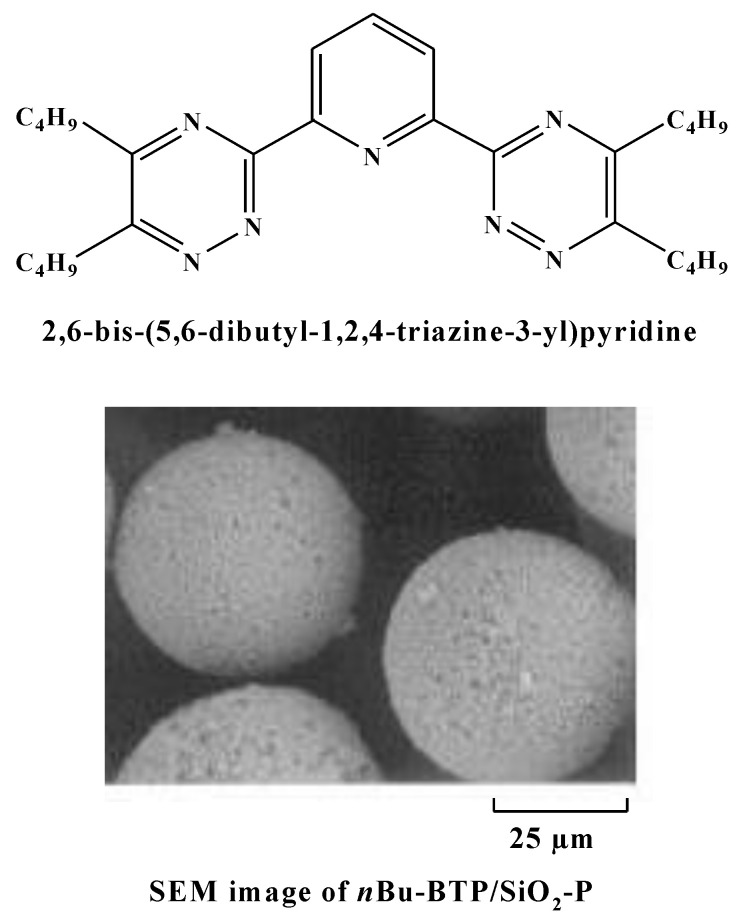
Structure and photograph of the *n*Bu-BTP/SiO_2_-P adsorbent.

As the support for the *n*Bu-BTP, the silica/polymer composite support (SiO_2_-P) developed in our previous work was utilized. It contains a macroreticular styrene–divinylbenzene copolymer that is immobilized in porous silica particles with a pore size of 0.6 µm and mean diameter of 60 µm. The *n*Bu-BTP was impregnated into the SiO_2_-P support at 33.3 wt% of Bu-BTP and the preparation procedure was described in our previous works [8,9]. The chemical structure of the *n*Bu-BTP and an SEM image of *n*Bu-BTP/SiO_2_-P adsorbent are illustrated in Figure 2. The *Iso*Bu-BTP/SiO_2_-P adsorbent was prepared using the vacuum impregnation method [2]. Typically, 5 g *iso*Bu-BTP dissolved in CH_2_Cl_2_ was impregnated into 10 g of SiO_2_-P under vacuum conditions using a rotary evaporator with a pressure controller.

Here, 2,6-bis-(5,6-dibutyl-1,2,4-triazine-3-yl)pyridine (*n*Bu-BTP or *iso*Bu-BTP) was synthesized in our laboratory with a purity level of 97% according to the high-performance liquid chromatography (HPLC) procedure (Prominence Plus, Shimadzu, Kyoto, Japan). The morphology and microstructure of the *n*Bu-BTP and *iso*Bu-BTP/SiO_2_-P adsorbents were characterized using scanning electron microscopy (SEM-EDS) (SU5000, HITACHI, Kyoto, Japan). The concentrations of stable Ln elements in the aqueous phase were determined using inductively coupled plasma atomic emission spectrometry (ICP-AES) (ICPS-7510, Shimadzu, Kyoto, Japan). The ^241^Am was measured using a high-purity germanium multichannel gamma spectrometer (GEM70P-PLUS, ORTEC, Oak Ridge, TN, USA), while ^244^Cm was determined using an alpha spectrometer (ALPHA-ENSEMBLE, ORTEC, Oak Ridge, TN, USA).

### 2.3. Batch Adsorption Experiment

Batch adsorption experiments were conducted to evaluate the adsorbability of Am(III) and some trivalent Ln fission products towards the *iso*Bu-BTP adsorbent. A determined volume (typically 5 cm^3^) of a sample solution containing 10 mM of each Ln(III) was spiked with the radioactive nuclides ^243^Am and ^152^Eu. A weighed amount of adsorbent (typically 0.25 g) was combined in a glass vial with a Teflon stopper with the sample solution. The resultant mixture was maintained in a water bath shaker kept at 298 K and mechanically shaken at 120 rpm until reaching an equilibrium state. After adsorption, the aqueous phase was filtrated through a membrane filter with 4.5 µm pores and sampled for quantitative measurements. The gamma activities of ^243^Am and ^152^Eu were detected using a γ-ray spectrometer with a high-purity germanium detector (Seiko EG&G). The average count rate was taken from three measured values to determine the activity. The concentration of stable Ln elements in the aqueous phase was analyzed using an ICP-AES instrument. The distribution coefficient (*K_d_*) was calculated using Equation (1). Typical experiments were performed in triplicate. The relative error rates of the adsorption experiments were all less than 5%.

(1) $$\begin{array}{ll} K_{d} & =\frac{C_{0}-C_{S}}{C_{S}}\times \frac{V_{S}}{W_{R}}\\ & =\frac{A_{0}-A_{S}}{A_{S}}\times \frac{V_{S}}{W_{R}}\;\left({\mathrm{dm}}^{3}/\mathrm{kg}\right) \end{array}$$

where *C*_0_ and *C_S_* denote the element concentration, while *A*_0_ and *A_S_* present the nuclide activity in the aqueous phase before and after adsorption, respectively. *W_R_* indicates the weight of the dry adsorbent (kg) and vs. the volume of the aqueous phase (dm^3^).

### 2.4. Hot Column Separation Experiment

The HLLW sample solution containing 6 M of HNO_3_ was generated from the anion exchange process that had separated the U, Pu, Pd, Ru, and Tc from a spent LWR fuel solution [12,16]. Prior to the separation of MA/Ln, the HLLW sample was fed to a CMPO/SiO_2_-P packed column for the elemental group partitioning according to the procedure shown in Figure 1. From this column, a genuine MA-Ln effluent containing Am, Cm, and almost all the FP lanthanides was obtained [12]. The solid-state NaNO_3_ was added into the MA-Ln effluent to the concentration of 0.3 M (M = mol/dm^3^). The pH value of the effluent was estimated to be around 1. Table 1 illustrates the analytic results of the representative nuclides contained in the sample solution.

In this work, we conducted a separation experiment for the genuine MA-Ln effluent using a Pyrex glass column with a 10 mm inner diameter and 300 mm length. The *n*Bu-BTP/SiO_2_-P adsorbent was filled to the column in a slurry state under 2–3 bars of nitrogen gas pressure. The temperature of the column and the throughput liquid phase were maintained constantly at 298 K. Before the chromatographic operation, the *n*Bu-BTP/SiO_2_-P packed in the column was conditioned by pumping 100 cm^3^ of 1 M NaNO_3–_0.01 M HNO_3_ solution through the column. Next, 30 cm^3^ of the sample solution was fed into the column at a constant flow rate of 1.0 cm^3^/min (0.76 m/h) using an HPLC metering pump. Subsequently, 70 cm^3^ of 0.3 M NaNO_3_ and 110 cm^3^ of H_2_O were supplied to the column as eluents, respectively. The outlet effluents from the column were collected using an auto-fractional collector in 10 cm^3^ aliquots. The concentrations and activities of the nuclides in each aliquot were quantitatively measured using ICP-MS and alfa/gamma spectrometry.

## 3. Results and Discussion

### 3.1. Adsorption and Desorption Behavior of Am(III) and Ln(III)

**Figure 3:**
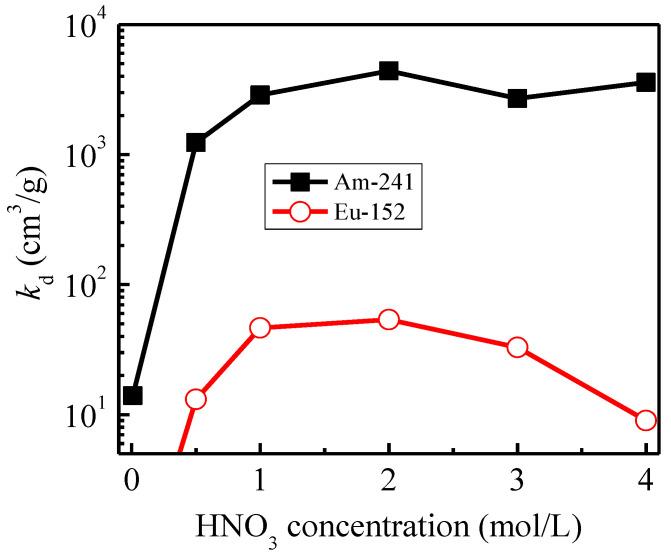
Effect of nitric acid concentration on the adsorption of ^241^Am(III) and ^152^Eu(III) onto *iso*Bu-BTP/SiO_2_-P (pH 2, 298 K, 24 h).

Figure 3 presents the distribution coefficients (K*_d_*) of ^241^Am(III) and ^152^Eu(III) towards the *iso*Bu-BTP/SiO_2_-P adsorbent with the change in the initial HNO_3_ concentration in the solution. The adsorption affinity of Am(III) increased drastically with the increase in HNO_3_ concentration and the *K_d_* reached near 5 × 10^3^ cm^3^/g in the range of 1–4 M HNO_3_. The K*_d_* of Eu(III) also increased with the increasing HNO_3_ concentration, but the adsorbability of Eu(III) was much lower than that of Am(III). The separation factor (SF_Am/Ln_ = ratio of K*_d_*-Am to K*_d_*-Eu) between Am(III) and Eu(III) is about 100 in the measured 0.5–4.0 M HNO_3_ range, which would be high enough for their efficient separation by means of the chromatography method.

Since Bu-BTP is a neutral ligand, the adsorption of trivalent metal cations from nitric acid or nitrate solution towards the adsorbent is supposed to result from the following complexing reaction [2]:

M^3+^_aq_ + 3NO_3_^−^_aq_ + 3(Bu-BTP)_ads_ = [M(NO_3_)_3_(Bu-BTP)_3_]_ads_ (2)

Because the stoichiometric ratio of M:NO_3_^−^ is 1:3, the adsorption ability of trivalent MA strongly depends on the concentration of nitrate ion (NO_3_^−^) surrounding the nitrogen ligand of the adsorbent (Figure 2). Nitric acid is a very strong acid, which almost completely liberates into a nitrate ion (NO_3_^−^) and proton (H^+^) in aqueous solutions. Therefore, the increase in adsorbability with HNO_3_ corresponds to the increase in NO_3_^−^ concentration. Notably, since the adsorption of Am(III) and Eu(III) drastically decreased when reducing the concentration of nitrate, and as they showed almost no adsorption in very dilute HNO_3_ solution, the adsorbed elements are expected to be eluted out when decreasing the NO_3_^−^ concentration in the chromatographic separation process. On the other hand, the mechanism for the soft donor ligands containing nitrogen or sulfur to exhibit a significantly higher adsorption affinity for trivalent actinides over lanthanides has not yet been clarified.

**Figure 4:**
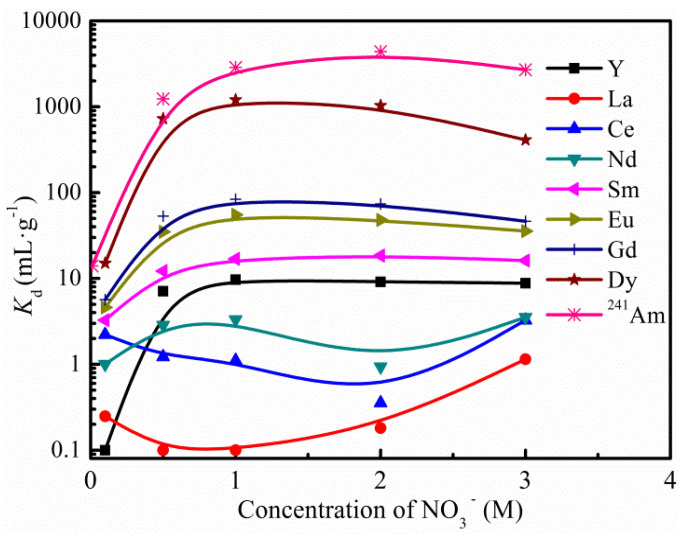
Relationship of adsorbability of ^241^Am(III) and Ln(III) towards *iso*Bu-BTP/SiO_2_-P with the nitrate concentration (NaNO_3_, pH 2, 298 K, 24 h).

The effects of the nitrate (NaNO_3_) concentration on the K*_d_* values of Am(III) and some trivalent lanthanides are shown in Figure 4. As can be seen, the adsorption ability of *iso*Bu-BTP/SiO_2_-P towards both ^241^Am(III) and Ln(III) increased with the NO_3_^−^ concentration, but the adsorbability of Am(III) is much higher than that of Ln(III). The separation factors between Am(III) and the Ln(III) are over 100 at a wide range of NO_3_^−^ concentrations, except SF_Am/Dy_. In addition, the adsorption of Ln(III) increased regularly with the atomic number and Dy(III) showed the strongest adsorption among the tested Ln(III) elements. In our previous work, we studied the coordination chemistry for the adsorption of some Ln(III) ions towards *iso*Bu-BTP/SiO_2_-P by means of an EXAFS analysis using the Ln(III)-adsorbed *iso*Bu-BTP/SiO_2_-P samples, e.g., Sm(III), Eu(III), Gd(III), and Dy(III) [2,5]. It was found that the [Ln(*iso*Butyl-BTP/SiO_2_-P)_3_]^3+^ complex is the dominant species and the Ln(III)-N bond length in the first coordination layer become shorter as the atomic number of Ln(III) increases, which indicates the enhanced interaction between Ln(III) and the *iso*Bu-BTP/SiO_2_-P adsorbent as the atomic number increases. Therefore, the relationship between adsorption affinity of *iso*Bu-BTP/SiO_2_-P and the different Ln(III) shown in Figure 4 can be reasonably explained by the EXAFS analysis results.

The lanthanide fission products resulting from the ^235^U fission reaction mostly include the light lanthanides from La to Tb, while the yield of Dy is almost negligible [17]. Since Dy(III) exhibits the strongest adsorbability among the lanthanides onto the R-BTP adsorbents and its adsorption behavior is similar to Am(III), it can be used as a simulated element of Am in some cases [18,19,20].

**Figure 5:**
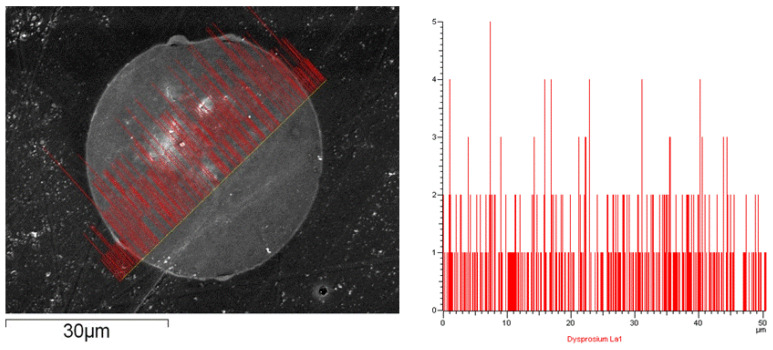
SEM-EDS of Dy(III)-loaded *iso*BTP/SiO_2_-P after batch adsorption for 1 h.

The SEM-EDS analysis result for the Dy-loaded isoBu-BTP/SiO_2_-P is shown in Figure 5. After the adsorption experiment, the Dy-loaded isoBu-BTP/SiO_2_-P adsorbent was inlaid into an epoxy resin. The particle size of the *iso*BTP/SiO_2_-P adsorbent is around 50 µm. As can be seen, the Dy(III) was adsorbed from the surface into the inside center of the particle and then distributed across the whole cross-section of the adsorbent. The images prove that the Dy(III) was evenly adsorbed inside the *iso*Bu-BTP/SiO_2_-P particles, not just on the surface.

**Figure 6:**
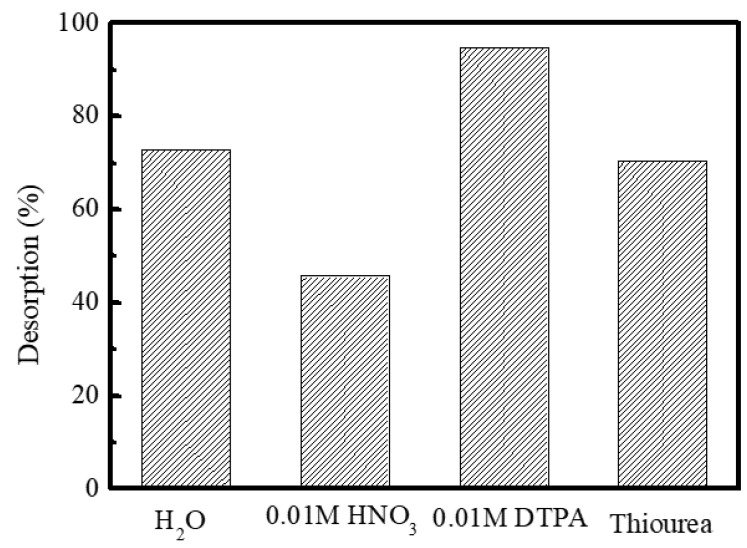
Effect of the eluting agent on the desorption rate of Dy(III) from the loaded *iso*Bu-BTP/SiO_2_-P (adsorption conditions: 298 K, phase ratio: 0.1 g/5 cm^3^, 120 rpm, 24 h).

To examine the desorption behavior of the trivalent metal ions from *iso*Bu-BTP/SiO_2_-P, batch desorption experiments were conducted using different eluting solutions. Figure 6 shows the effect of the eluting agent on the desorption rate of the Dy(III) from the loaded *iso*Bu-BTP/SiO_2_-P adsorbent. As can be seen, DTPA(diethylenetriaminepentaacetic acid) exhibited highest desorption rate of the Dy(III) ions. DTPA is an aqueous soluble nitrogen bearing a complexing agent and can coordinate with Dy(III), resulting in its desorption from the adsorbent. On the other hand, the adsorbed Dy(III) was quite effectively eluted out by the water due to a drastic decrease in the nitrate concentration in the adsorbent.

### 3.2. Chromatographic Separation of Am(III) from Ln(III)

**Figure 7:**
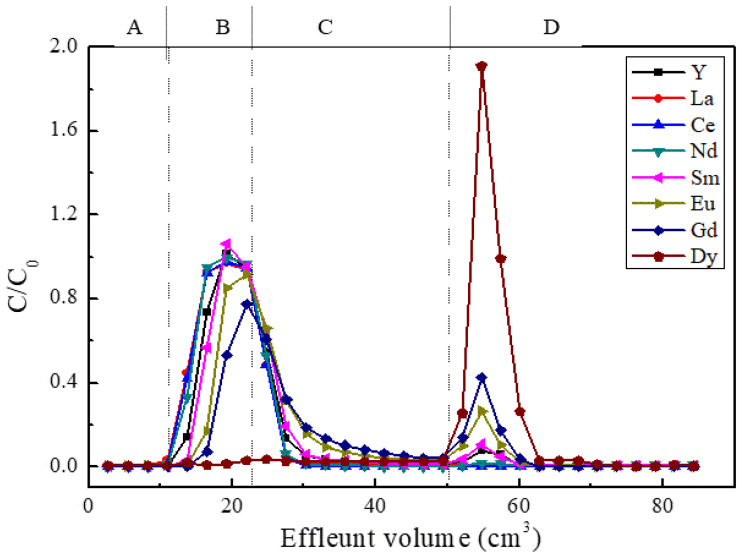
Column dynamic adsorption–elution experiment results for a Ln(III) mixed sample solution using *iso*Bu-BTP/SiO_2_-P adsorbent (column: ø10 mm × *h*300 mm; flow rate: 0.3 mL/min; temperature: 298 K): (**A**) washing solution (0.01 M HNO_3_–0.49 M NaNO_3_); (**B**) feed sample solution; (**C**) rinsing solution (0.01 M HNO_3_–0.49 M NaNO_3_); (**D**) 0.01 M DTPA.

Prior to the hot test, a column dynamic adsorption–elution experiment using the *iso*Bu-BTP/SiO_2_-P adsorbent was carried out for a mixed Ln(III) sample solution with Dy(III) as an analogue of Am(III). The feed sample contained 1 mM of Y(III), La(III), Ce(III), Nd(III), Sm(III), Eu(III), Gd(III), and Dy(III) in a 0.01 M HNO_3_–0.49 M NaNO_3_ solution, respectively. The elution curves of the Ln(III) ions are shown in Figure 7. As can be seen, the light Ln(III) ions, such as La(III), Ce(III), Nd(III), and Y(III), showed almost no adsorption and flowed out from the column with the feed sample and the rinsing solution. The Dy(III) was completely adsorbed and was effectively eluted off by 0.01 M DTPA as eluent. Small portions of Sm(III), Eu(III), and Gd(III) were adsorbed and were eluted together with Dy(III). It was found that the column dynamic adsorption–elution results show good agreement with the batch adsorption–desorption experiments.

**Figure 8:**
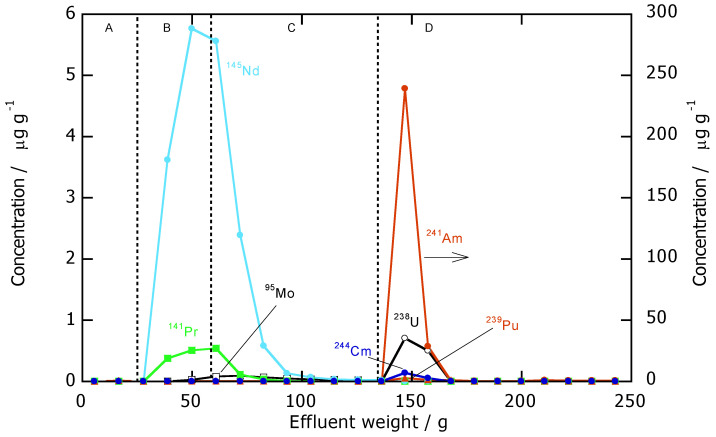
Separation experiment results for the genuine MA-Ln effluent by *n*Bu-BTP/SiO_2_-P packed column: (**A**) dead volume; (**B**) sample solution; (**C**) 0.3 M NaNO_3_; (**D**) H_2_O.

In our previous work, a hot test for an actual high-level liquid waste (HLLW) generated from an irradiated MOX fuel treatment process was carried out using a CMPO/SiO_2_-P adsorbent packed column. As a result, Am and Cm together with the fission product Ln were successfully separated from other FPs contained in the HLLW, resulting in an MA-Ln effluent with a pH of around 1 [12]. In this work, this weakly acidic MA-Ln effluent was adjusted to 0.3 M of NaNO_3_ and then subjected to a *n*Bu-BTP/SiO_2_-P packed column for Am/Ln separation. Figure 8 shows the results of the separation experiment for the genuine MA-containing effluent with the nuclide composition, as shown in Table 1. As can be seen, the Ln(III) represented by Nd(III) and Pr(III) showed almost no adsorption. They flowed out with the sample solution and the 0.3 M NaNO_3_ rinsing solution. On the other hand, Am(III) and Cm(III) were strongly adsorbed by the *n*Bu-BTP/SiO_2_-P adsorbent, and then were effectively eluted off using pure water as an eluent. A complete separation between MA (Am and Cm) and the Ln was achieved.

In the sample solution, small amounts of U, Pu, and Mo were contained. The elution profiles of these nuclides together with the MA and Ln are illustrated in Figure 8. It was found that U and Pu were also adsorbed by the adsorbent and efficiently eluted off by H_2_O together with the MA. Since the spent fuel solution was once reduced by electro-reduction prior to the anion exchange separation process, the valence representations of U and Pu in the MA-Ln effluent were considered to be U(IV) and Pu(IV) or Pu(III), respectively [16]. From this experimental result, we can conclude that the U and Pu that remained in MA-Ln effluent could be effectively recovered together with the MA. On the other hand, the Mo showed very weak adsorption and behaved similarly to the non-adsorptive Ln(III).

These experimental results demonstrate that the proposed MA separation process shown in Figure 1 is feasible in principle. For future practical applications, further investigations including improvements of the radiation stability of the adsorbents and scale-up testing are required.

## 4. Conclusions

The adsorption and desorption behavior of Am(III) and Ln(III) onto Bu-BTP/SiO_2_-P adsorbent were investigated. Column separation experiments using simulated and genuine MA effluent were carried out. The *iso*Bu-BTP/SiO_2_-P exhibited much stronger adsorbability for ^241^Am(III) than ^152^Eu(III) and all of the Ln(III) ions in the acidic nitrate solution. The separation factor between Am(III) and Ln(III)-FP was over 100 in the measured 0.5–4 M NaNO_3_ solution with a pH of 2. The adsorbed Am(III) and Ln(III) ions were effectively eluted out using DTPA or pure water as the eluents. The column experiment results demonstrated that the Ln(III)-FP showed almost no adsorption, while the Am(III) and Cm(III), as well as the residual U and Pu, were completely adsorbed by the adsorbent and eluted off by using water as an eluent. A complete separation between the MA and Ln was achieved, indicating that the proposed MA separation process is feasible in principle. Further investigations including improvements of the radiation stability of the adsorbents and scale-up testing are required.

## Figures and Tables

**Table 1:** Analytic results of the genuine MA effluent used for the separation experiment.

Nuclide	Concentration	Analysis
	(µg/g-soln)	Method
^95^Mo	0.2	ICP-MS
^141^Pr	0.7	ICP-MS
^145^Nd	10.9	ICP-MS
Nd-total	62.9	ICP-MS
^238^U	6.7	ICP-MS
^239^Pu	0.2	ICP-MS
^241^Am	84.4	γ spectrometry
^244^Cm	0.1	α spectrometry

## Data Availability

Not applicable.

## References

[1] Zhang W., He X., Ye G., Yi R., Chen J. (2014). Americium(III) capture using phosphonic acid-functionalized silicas with different mesoporous morphologies: Adsorption behavior study and mechanism investigation by EXAFS/XPS. Environ. Sci. Technol..

[2] Ning S.Y., Zhang W., Yu S.Q., Zhang S.C., Zhou J., Wang X.P., Wei Y.Z. (2019). Selective separation of MA(III) from Ln(III) by highly stable silica-polymer based N-donor IsoBu-BTP/SiO_2_-P adsorbent. Solvent Extr. Ion Exch..

[3] Madic C. Overview of the hydrometallurgical and pyro-metallurgical processes studied world-wide for the partitioning of high active nuclear wastes. Proceedings of the NEA/OECD 6th Information Exchange Meeting on Actinide and Fission Product Partitioning and Transmutation.

[4] Ning S.Y., Zhang S.C., Zhou J., Zhang W., Wei Y.Z. (2019). Salt-free separation of ^241^Am(III) from lanthanides by highly stable macroporous silica-polymer based Me_2_-CA-BTP/SiO_2_-P adsorbent. J. Radioanal. Nucl. Chem..

[5] Ning S.Y., Zou Q., Wang X.P., Liu R.Q., Wei Y.-Z. (2016). Adsorption mechanism of silica/polymer-based 2,6-bis(5,6-diisohexyl-1,2,4-triazin-3-yl)pyridine adsorbent towards Ln(III) from nitric acid solution. J. Nucl. Sci. Technol..

[6] Kolarik Z., Müllich U., Gassner F. (1999). Selective extraction of Am(III) over Eu(III) by 2,6-ditriazolyl-and 2,6-ditriazolylpyridines. Solvent Extr. Ion Exch..

[7] Kolarik Z., Müllich U., Gassner F. (1999). Extraction of Am(III) and Eu(III) nitrates by 2,6-Di-(5,6-dipropyl-1,2,4-triazine-3-yl). Solvent Extr. Ion Exch..

[8] Wei Y.-Z., Kumagai M., Takashima Y., Modolo G., Odoj R. (2000). Studies on the separation of minor actinides from high-level wastes by extraction chromatography using novel silica-based extraction resins. Nucl. Technol..

[9] Wei Y.-Z., Kumagai M., Takashima Y., Yokoi H., Hoshikawa T., Kawamura F. Development of silica-based chelating exchangers and their separation behavior for lanthanides and americium by column chromatography. Proceedings of the RECOD’98.

[10] Zhang H., Li C.M., Chen X.J., Fu H., Chen Y.L., Ning S.Y., Fujita T., Wei Y.Z., Wang X.P. (2022). Layered ammonium vanadate nanobelt as efficient adsorbents for removal of Sr^2+^ and Cs^+^ from contaminated water. J. Colloid Interf. Sci..

[11] Wei Y., Ning S., Wang Q., Chen Z., Wu Y., Liu R., Mimura H. (2014). Adsorption materials development for the separation of actinides and specific fission products from high level waste. Adv. Sci. Technol..

[12] Wei Y.-Z., Hoshi H., Kumagai M., Goethals P., Bruggeman A., Alvares R., Bryan N., May I. (2006). A hot test on minor actinides separation from high-level-waste by CMPO/SiO_2_-P extraction resin. Recent Advances in Actinide Science.

[13] Wei Y.-Z., Sabharwal K.N., Kumagai M., Asakura T., Uchiyama G., Fujine S. (2000). Preparation of novel silica-based nitrogen donor extraction resins and their adsorption performance for trivalent americium and lanthanides. J. Nucl. Sci. Technol..

[14] Wei Y.-Z., Hoshi H., Kumagai M., Asakura T., Uchiyama G. (2002). Preparation of novel silica-based R-BTP extraction-resins and their application to trivalent actinides and lanthanides separation. J. Nucl. Sci. Technol..

[15] Wei Y.-Z., Hoshi H., Kumagai M., Asakura T., Morita Y. (2004). Separation of Am(III) and Cm(III) from trivalent lanthanides by 2,6-bistriazinylpyridine extraction chromatography for radioactive waste management. J. Alloys Compd..

[16] Wei Y.-Z., Arai T., Hoshi H., Kumagai M., Bruggeman A., Goethals P. (2005). Development of a new aqueous process for nuclear fuel reprocessing: Hot tests on the recovery of U and Pu from a nitric acid solution of spent LWR fuel. Nucl. Technol..

[17] Benedict M., Pigford T.H., Levi H.W. (1981). Nuclear Chemical Engineering.

[18] Geist A., Hill C., Modolo G., Foreman M.R.S.J., Weigl M., Gompper K., Hudson M.J. (2006). 6,6′-Bis(5,5,8,8-tetramethyl-5,6,7,8-tetrahydro-benzo [1,2,4]triazin-3-yl) [2.;2′]bipyridine, an effective extracting agent for the separation of americium(III) and curium(III) from the lanthanides. Solv. Extr. Ion Exch..

[19] Trumm S., Geist A., Panak P.J., Fanghänel T. (2011). An improved hydrolytically-stable bis-triazinyl-pyridine (BTP) for selective actinide extraction. Solv. Extr. Ion Exch..

[20] Usuda S., Wei Y.-Z., Xu Y., Li Z., Liu R., Kim S.-Y., Wakui Y., Hayashi H., Yamazaki H. (2012). Development of a simplified separation process of trivalent minor actinides from fission products using novel R-BTP/SiO_2_-P adsorbents. J. Nucl. Sci. Technol..

